# Chronic Chikungunya Arthritis in Northeastern Brazil: An Association with Very Severe Joint Pain and Lack of Correlation with *IL-6* and *TNFα* Gene Polymorphisms

**DOI:** 10.3390/v17121543

**Published:** 2025-11-26

**Authors:** Mariella Sousa Coêlho Maciel, Catharina Diniz de Brito Martins, Alan Gleison Moreira dos Santos, Caroline Nobre Oliveira, Hygor Ferreira Fernandes, Raphael de Oliveira Rodrigues, Juliana Navarro Ueda Yaochite

**Affiliations:** 1 Laboratório de Imunologia Celular e Molecular (LIMCEMO), Departamento de Análises Clínicas e Toxicológicas, Universidade Federal do Ceará, Fortaleza 60430-370, CE, Brazil; mariellamaciel@hotmail.com (M.S.C.M.);; 2 Instituto Federal de Educação, Ciência e Tecnologia do Piauí, Parnaíba 64202-020, PI, Brazil

**Keywords:** arthritis chronic chikungunya, interleukin-6, tumor necrosis factor alpha, polymorphisms

## Abstract

Chikungunya fever (CHIKF) affects thousands of people annually. Therefore, aspects involved in the pathogenesis of the disease must be further explored. In this study, we assessed the development of chronic chikungunya arthritis (CCA) and the single-nucleotide polymorphisms (SNPs) -174 G/C in the interleukin-6 (*IL-6*) gene and -308 G/A in the tumor necrosis factor alpha (*TNFα*) gene on CHIKF development in a population from Northeastern Brazil. A total of 313 blood and serum samples were collected. Of these, 102 were included in the cases group, 182 were included in the control group, and 29 were incorporated in the asymptomatic group. The DNA extraction and genotyping across real-time quantitative polymerase chain reaction (RT-qPCR) were performed. The data showed that 71.6% of individuals who had CHIKF developed CCA. A significant increase in CCA development was observed in females, and very severe joint pain was associated with increased risk of CCA. In contrast, we did not identify significant associations between the SNPs and CHIKF development. Our data indicate that females and individuals who develop very severe joint pain have an increased risk for CCA and highlight the lack of correlation of SNPs in the *IL-6* and *TNFα* genes with CHIKF in the studied population.

## 1. Introduction

Chikungunya fever (CHIKF) is a disease caused by the *Alphavirus chikungunya* (CHIKV), which belongs to the genus *Alphavirus* within the family *Togaviridae* and is responsible for causing an important rheumatic condition [1,2]. The infection caused by CHIKV can range from asymptomatic to severe cases. However, most individuals develop symptoms, with arthralgia being the most characteristic clinical manifestation of CHIKV infection [3,4,5]. The persistence of arthralgia for more than three months indicates the development of a chronic phase, known as chronic chikungunya arthritis (CCA), which can persist for months or years [6,7,8]. However, differential diagnosis should be carefully considered to rule out other rheumatic diseases.

Since the identification of CHIKV to the current day, several epidemics have been triggered worldwide, resulting in a high morbidity rate [9,10]. On a global level, thousands of chikungunya cases are reported each year [11,12]. In Brazil, the first case reported was in 2014, in the North. However, between 2016 and 2017, large-scale outbreaks occurred in different parts of the country, where the Northeastern region was one of the most affected [13,14]. Although chikungunya cases have been reported in subsequent years, in 2023, the disease returned to the spotlight after an increasing number of cases in the Americas [15,16].

Due to the significant public health concern caused by CHIKF, there is a need for a greater understanding of the aspects surrounding the disease. Thus, the search for the identification of risk factors, as well as the characterization of the clinical aspects, plays an important role, as it can contribute to the development of new therapeutic strategies. Additionally, the analysis of the presence of single-nucleotide polymorphisms (SNPs) in cytokine genes can provide valuable insights into susceptibility or protection against the disease [17,18].

In this context, interleukin-6 (IL-6) has been detected in high concentrations in individuals with CHIKF, both in the acute and chronic phases [19,20,21]. In contrast, studies have shown that tumor necrosis factor alpha (TNFα) has been detected in lower concentrations in CHIKF patients during the acute stage, with greater secretion in individuals with persistent arthralgia [22,23]. Analyses of the SNPs -174 G/C of the *IL-6* gene and -308 G/A of the *TNFα* gene have been described as important factors capable of influencing the pathogenesis of rheumatoid arthritis (RA) [24,25]. Furthermore, these SNPs have also been studied in relation to the development of dengue and other viral diseases, including, more recently, chikungunya [26,27,28,29], which highlights the importance of this type of study in different populations, since genetic variability among groups can result in distinct profiles of frequency and influence of SNPs, as well as the absence of correlation of these with the development of infectious diseases [30].

This study provides evidence regarding the factors associated with the development of CCA and the long-term impact of arthralgia on the quality of life of those diagnosed with CHIKF. Moreover, it describes results inherent to the analyses of the SNPs -174 G/C of the *IL-6* gene and -308 G/A of the *TNFα* gene in a cohort of individuals who had chikungunya from Northeastern Brazil. Our research contributes to the understanding of the pathogenesis of chikungunya and of human genetics and its variability among different world populations.

## 2. Materials and Methods

### 2.1. Ethics

The Ethics and Research Committee of the Universidade Federal do Ceará, Fortaleza, Ceará, Brazil, approved this study with the number 3.212.740. All participants provided written informed consent prior to their involvement and were assured that their participation was voluntary, with the freedom to withdraw at any time without any consequences.

### 2.2. Study Population, Samples, and Clinical Data

This retrospective case–control study was conducted from March 2019 to June 2021 in the Laboratório de Imunologia Celular e Molecular at the Universidade Federal do Ceará, Fortaleza, Ceará, Northeastern Brazil. The recruitment of volunteers to compose the research was carried out randomly among the patients who sought care in the Laboratório de Análises Clínicas e Toxicológicas, also located at the Universidade Federal do Ceará. The inclusion criteria applied were individuals aged ≥18 years and residents in the state of Ceará, regardless of gender, race, or socioeconomic level.

The case group was composed of individuals who reported having developed at least three clinical signs and/or symptoms suggestive of CHIKF, having been affected by the disease between the years 2014 (the year of the first CHIKF case reported in Brazil) and 2019 (the year in which the recruitment of volunteers ended). Importantly, all patients included in the case group tested positive for anti-CHIKV IgG, and those who reported persistent joint pain lasting more than three months were classified as having CCA. No patient presented any clinical signs or symptoms suggestive of acute infectious disease at the time of blood collection. The control group consisted of individuals who reported never having experienced symptoms characteristic of any arboviral disease without a confirmed laboratory diagnosis for CHIKV infection. Individuals who reported no history of CHIKF but tested positive for anti-CHIKV IgG antibodies were classified into the asymptomatic group. Venipuncture was performed to collect blood and serum samples from each volunteer. Samples were centrifuged, aliquoted, and stored at a temperature of −20 °C.

Clinical data from patients were obtained through direct interviews using a semi-structured questionnaire, which included information on the patients’ sociodemographic and clinical variables, including onset of symptoms and the intensity of joint pain. The determination of joint pain parameters in this study was based on the verbal rating scales (VRS) [31], where individuals who had CHIKF were able to classify arthralgia as mild, moderate, severe, and very severe.

### 2.3. Enzyme-Linked Immunosorbent Assay

To confirm chikungunya cases, as well as to screen individuals in the control group and identify those asymptomatic, serological assays were performed to detect the presence of anti-CHIKV IgG antibodies. For this purpose, a commercial enzyme-linked immunosorbent assay (ELISA) (Euroimmun ^®^, Lübeck, Germany) was used, according to the manufacturer’s instructions.

### 2.4. DNA Extraction

Deoxyribonucleic acid (DNA) was extracted from 200 μL of whole blood using the Mini Spin Plus (BioPur ^®^, Paraná, Brazil) following the manufacturer’s instructions. After extraction, a 1 μL sample of DNA was quantified in duplicate or triplicate using the equipment NanoDrop One (Thermo Fisher Scientific ^®^, MA, USA) to verify the concentration and purity of the genetic material obtained.

### 2.5. Genotyping

Polymorphisms in the *IL-6* gene (-174 G/C—rs1800795) and *TNFα* gene (-308 G/A—rs1800629) were genotyped using real-time quantitative polymerase chain reaction (RT-qPCR). Validated SNP TaqMan ^®^ Genotyping Assays (Applied Biosystems, Waltham, MA, USA), Master Mix TaqPath ™ ProAmp ™ (Applied Biosystems, Waltham, MA, USA), and SNP detection probes were used for the *IL-6* and *TNFα* variants. The procedure was performed using the thermocycler CFX 96 ^TM^ Real Time System (Bio-Rad, Berkeley, CA, USA) according to the manufacturer’s protocol.

The thermal cycling conditions consisted of a pre-reading at 60 °C for 30 s and initial activation of the denaturing enzyme at 95 °C for 5 min, followed by 40 cycles of denaturation at 95 °C for 15 s and annealing and extension at 60 °C for 1 min. The post-reading step was performed at 60 °C for 30 s, ending the amplification procedure.

### 2.6. Data Analysis

The analyses and graphical representations were performed using GraphPad Prism version 8.0.2 (GraphPad software, San Diego, CA, USA). Comparisons between clinical and sociodemographic variables were performed using Fisher’s exact test. The Mann–Whitney test was used for comparisons between two groups in unpaired sample analyses. Regarding SNP analyses, the genotype and allele frequencies were determined using simple direct counting. To test the Hardy–Weinberg equilibrium, the genotype and allele distributions were compared using the chi-square test (χ^2^). The statistical significance of the comparisons between the cases, control, and asymptomatic groups was estimated using the chi-square test (χ^2^) and Fisher’s exact test, the latter being used when a value in the contingency table was less than 5.

An association strength or odds ratio (OR) was calculated for each comparison, considering OR > 1 associated with a risk factor, OR < 1 associated with protection, and OR = 1 indicating a balance between risk and protection, that is, no association. In all cases, a significance level of *p* < 0.05 was considered statistically significant.

## 3. Results

A total of 313 individuals were included in this study. Of these, 102 subjects with symptoms suggestive of CHIKF tested positive for anti-CHIKV IgG antibodies and were included in the case group; another 182 tested negative and were included in the control group. The asymptomatic group (ASY) consisted of 29 individuals initially recruited into the control group who later tested positive for anti-CHIKV IgG antibodies. The average age of the case group was 57.5 years (range: 18–86 years), with the majority being female (69.6%). For the control group, the average age was 47.8 years (range: 19–86 years), the majority being female (72%). The ASY group had an average age of 54.9 years (range: 18–88 years), with the majority being male (51.7%).

The frequency of clinical manifestations was evaluated among individuals in the case group. Through the analysis, it was possible to observe that arthralgia (100%), followed by fever (85.3%), myalgia (85.3%), headache (71.6%), and edema (63.7%), were the most predominant symptoms (Table 1).

**Figure 1:**
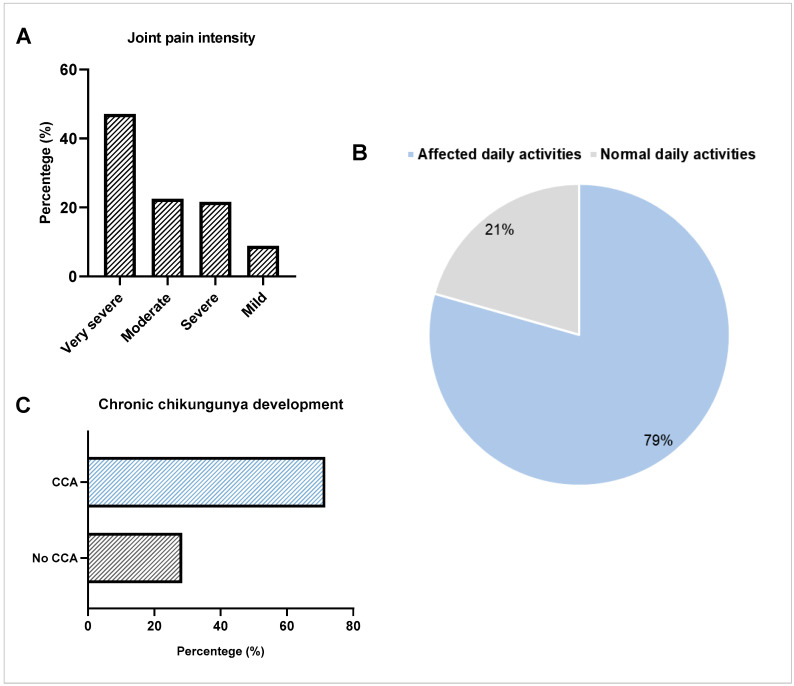
Outcome of arthralgia in individuals with chikungunya fever: (**A**) classification of joint pain intensity; (**B**) percentage of individuals with impaired physical activity due to arthralgia; (**C**) development of chronic chikungunya arthritis (CCA) in the study population.

Arthralgia was present in 100% of the individuals in the case group. The most affected joints were knees (75.5%), ankles (69.6%), and elbows (65.7%); less frequently affected were the shoulders (52.9%), wrists (49%), and hips (40.2%). Through four parameters previously established to assess the intensity of joint pain from reports of each individual, we observed that very severe (47.1%) and moderate (22.5%) pain were the most frequent, followed by severe (21.6%) and mild (8.8%) pain (Figure 1A). A total of 81 individuals (79%) considered that their daily activities were impaired due to CHIKF, whereas the other 21 volunteers (21%) reported that their daily activities were not affected (Figure 1B). Tasks reported as being impaired due to pain ranged from domestic activities, such as sweeping the floor and washing dishes, to the performance of professional duties.

In addition, we assessed the number of individuals who developed CCA and observed that among the 102 individuals who had CHIKF, a total of 73 (71.6%) developed CCA (Figure 1C), with an average age of 58.1 years, ranging from 28 to 86 years, the majority being female (76.7%). The median age was 57 years [interquartile range: 52–68] in the CCA group (*n* = 73) and 61 years [interquartile range: 36.50–68.50] in the non-CCA group (*n* = 29). The difference was not statistically significant by the Mann–Whitney test (*p* = 0.983). The duration of joint pain ranged from months to years, with one case identified in which arthralgia lasted for up to 5 years after the acute phase.

To identify possible associations between sex and joint pain intensity with the development of CCA, these variables were compared between individuals who did and did not develop the chronic phase of chikungunya. Through the analyses, it was observed that females (76.7%) had a higher frequency of developing CCA compared to males (23.3%), presenting an increased risk of developing the condition (*p* = 0.018; OR = 3.08). Regarding joint pain intensity, we identified that mild pain was more frequent in the non-CCA group (20.7%) and was associated with a lower risk of developing CCA (*p* = 0.015; OR = 0.16). Moderate pain was more frequent in the non-CCA group (37.9%) and was also associated with a lower risk of developing CCA (*p* = 0.034; OR = 0.32). In contrast, very severe pain was more frequent in the CCA group (58.9%) and was significantly associated with an increased risk of CCA (*p* < 0.001; OR = 6.88). No statistically significant association was observed for severe pain (*p* = 0.791; OR = 0.81) (Table 2).

Regarding SNPs, the genotypic and allelic analyses were performed to obtain clarifications about the presence and influence of the variants -174 G/C of the *IL-6* gene and -308 G/A of the *TNFα* gene under the CHIKF development in this study population. All samples were genotyped, and all genotypic frequencies investigated for both SNPs were found in Hardy–Weinberg equilibrium. The analysis of the genotypic frequencies obtained for the SNP -174 G/C of the *IL-6* gene revealed that there was a higher frequency of the G/G genotype in the case group (54.9%) compared to the control group (47.8%). In contrast, the heterozygous G/C genotype was more frequent in the control group (43.9%) than in the case group (38.2%), and the C/C homozygote was more frequent in the control group (8.2%) compared to the case group (6.9%). However, there was no statistical significance (*p* = 0.284; OR = 0.76; *p* = 0.509; OR = 0.72, respectively).

Genotype frequencies (G/C + G/G and G/C + C/C) did not show statistically significant differences between the case and control groups (*p* = 0.563; OR = 0.88; *p* = 0.250; OR = 0.75, respectively). In the analyses of allelic frequencies, the G allele was more frequent in the case group (74.0%) than in the control group (69.8%), while the C allele was more frequent in the control group (30.2%) compared to the case group (26.0%) (*p* = 0.283; OR = 0.81), with no statistical significance (Table 3).

The genotypic frequency analyses of the SNP -174 G/C of the *IL-6* gene between the groups ASY and case showed that the frequency of the G/G genotype was higher in the ASY group (58.6%) compared to the case group (54.9%), while the frequency of the G/C genotype was higher in the case group (38.2%) compared to the ASY group (37.9%), with no statistical significance (*p* = 0.867; OR = 0.93). The comparison of the genotypes G/C + G/G and G/C + C/C between the ASY and case groups does not reveal statistical significance (*p* = 0.932; OR = 0.97; *p* = 0.722; OR = 0.86, respectively).

Regarding the analyses of allele frequencies, the G allele was more frequent in the ASY group (77.6%) compared to the case group (74.0%), while the C allele was more frequent in the case group (26.0%) compared to the ASY group (22.4%). However, such analyses did not reveal a statistically significant value (*p* = 0.580; OR = 0.82). Similarly, for genotypic and allelic comparisons between the ASY and control groups, no statistically significant differences were observed (Table 4).

The analysis of the genotypic frequencies for the SNP -308 G/A in the *TNFα* gene showed a higher frequency of the G/G genotype in the control group (78.0%) compared to the case group (71.6%). Moreover, the heterozygous G/A genotype exhibited a higher frequency in the case group (28.4%) compared to the control group (21.4%), and the A/A genotype was absent in individuals from the case group, being observed only in the control group of the study (0.5%). No statistically significant difference was observed (*p* = 0.193; OR = 1.45; *p* = 1.000; OR = 0.00, respectively).

Through comparisons of the frequencies of the G/A + G/G genotypes, it was possible to observe a greater presence in the case group (100%) compared to the control group (99.4%), and for comparisons between the G/A + A/A genotypes, the case group had a higher frequency (28.4%) compared to the control group (22.0%), without statistical significance (*p* = 0.628; OR = 1.10; *p* = 0.223; OR = 1.41, respectively). The allelic analyses revealed a higher frequency of the G allele in the control group (88.7%) compared to the case group (85.8%). The A allele had a higher frequency in the case group (14.2%) compared to the control group (11.3%). However, no statistically significant value was observed (*p* = 0.304; OR = 1.31) (Table 5).

In order to complement the data, the analysis of the genotypic frequencies was performed for the SNP -308 G/A in the *TNFα* gene between the groups ASY and case, as well as ASY and control. Thus, the frequency of the G/G genotype was higher in the ASY group (79.3%) compared to the case group (71.6%), while the frequency of the G/A genotype was higher in the case group (28.4%) compared to the ASY (20.7%), with no statistical significance (*p* = 0.405; OR = 0.66). The comparison between the genotypes G/A + G/G and G/A + A/A between the ASY and case groups does not reveal statistical significance (*p* = 0.747; OR = 0.90; *p* = 0.405; OR = 0.66, respectively).

Regarding the analysis of allele frequencies, the G allele was more frequent in the ASY group (89.6%) compared to the case group (85.8%). The A allele was more frequent in the case group (14.2%) compared to the ASY group (10.3%). However, with no statistical significance (*p* = 0.444; OR = 0.70). Similarly, for genotypic and allelic comparisons between the ASY and control groups, no statistically significant differences were observed (Table 6).

Allelic analysis between carriers and non-carriers of the mutant C and A alleles of SNPs in the *TNFα* and *IL-6* genes, respectively, for individuals with different intensities of joint pain did not reveal significant associations (Supplementary Tables S1 and S2). Significant differences also were not observed for the analysis of SNPs in the *TNFα* and *IL-6* genes in relation to comparisons involving individuals with chronic chikungunya (Supplementary Tables S3 and S4).

## 4. Discussion

The data showed that all individuals who had CHIKF developed arthralgia, making evident the arthritogenic nature of CHIKF. Moreover, other signs and symptoms reported more frequently were fever, myalgia, and headache, similar to those observed in other studies worldwide [32,33,34]. Notably, most individuals reported very severe joint pain, and 79% of the study population considered their daily activities impaired due to arthralgia. This finding consolidates existing evidence regarding the impact of CHIKF on the quality of life of those affected [10,35,36].

In this research, we found that 71.6% of individuals developed CCA; in one case reported, arthralgia lasted for up to 5 years after the acute phase. Previously, a study conducted by Ninla-Aesong et al. [20] had demonstrated that patients with persistent joint manifestations 5 years after the acute phase of CHIKF. Additionally, other studies have reported a prolonged duration of CCA [32,37], which also results in an economic burden due to medical treatment costs [38].

In this study, we observed that female sex and very severe joint pain were significantly associated with an increased risk of CCA development. Previous studies have demonstrated that advanced age, female sex, and severe pain in the acute stage may contribute to the development of CCA [39,40,41]. Our research contributes to the understanding of the pathogenesis of chikungunya and consolidates existing evidence on risk factors for CCA, particularly female sex and very severe joint pain.

Cytokine gene SNPs are commonly associated with the protection or progression of infectious diseases, as has been evidenced in cases of infection by CHIKV and other arboviruses [17,18,42]. In this study, it was not possible to find significant evidence for the comparisons made between the groups that composed the research in relation to SNPs -174 G/C of the *IL-6* gene and -308 G/A of the *TNFα* gene with the development of CHIKF. However, carrying out genetic studies involving the investigation of SNPs contributes not only to the identification of protective factors or susceptibility to pathologies but also to the understanding of human genetics and its variability among different world populations, corroborating better elucidations about biological phenomena.

To date, there is not a range of studies with SNPs analyses aimed at a better understanding of the pathogenesis of CHIKF. Therefore, it is necessary to correlate the results shown in the present study with those obtained from research involving analysis of the variants in the *TNFα* and *IL-6* genes intended for other diseases, including rheumatoid arthritis and dengue, which have similar characteristics to CHIKF.

The SNP -174 G/C of the *IL-6* gene has been reported in the literature as being capable of influencing the expression of this pro-inflammatory cytokine, which exerts immunoregulatory and pleiotropic effects. The C allele of this SNP is mentioned to result in lower levels of IL-6, while the G allele is known as the common allele [43,44]. Although no significant associations with respect to this SNP were observed in this study, the data obtained through this research showed that the C allele, referred to as polymorphic, was more frequent in the control group in relation to the case, as well as the C/C genotype, which was more frequent in the control group of the study.

In a study with analysis of SNPs for RA, developed by Amr et al. [24] in an Egyptian population, it was observed that the C allele of the SNP -174 G/C in the *IL-6* gene was more frequent in the group of individuals with RA, as well as the G/C and C/C genotypes were more frequent in this same group compared to the control; such differences were statistically significant, with the presence of the C allele associated as a risk factor for the disease. In contrast, Pawlik et al. [45] in a study carried out in a Polish population, significantly associated the presence of the G/G genotype of the SNP -174 G/C as a factor of risk for RA. It was observed that in patients homozygous for the G allele, the course of the disease was more severe compared to those with the C allele.

In a study developed by Moreira et al. [46] in a Brazilian population in the state of Paraná, a significant association was reported between the presence of the heterozygous genotype G/C of the SNP -174 G/C in the *IL-6* gene as a protective factor against the development of dengue fever, with this genotype present more frequently in the control group compared to the case group. In addition, in a study performed by Queiroz et al. [29] in Northeastern Brazil, a significantly higher frequency of the C allele of the SNP -174 G/C was evidenced in asymptomatic individuals compared to individuals with confirmed dengue infection, with the presence of this allele associated as a protective factor against the disease. For chikungunya, there were no statistically significant differences. However, the C allele was also more frequent in individuals in the control group, the latter result being similar to that obtained in the present study.

The SNP -308 G/A of the *TNFα* gene may also influence the expression of this important pro-inflammatory cytokine, which participates in different immunological processes. The findings contained herein reveal that the A allele of this gene, known as polymorphic, was present at a lower frequency in the general study population compared to the G allele, mentioned as the common allele. Several studies involving the analysis of SNPs, including the -308 G/A variant of the *TNFα* gene, were conducted in different countries. However, correlations between genotypes of this gene with joint damage remain divergent [47,48].

Rizvi et al. [49] in a study investigating SNPs for young-onset RA in a population in Pakistan, found that there was an absence of the A/A genotype of the SNP -308 G/A of the *TNFα* gene for the control group, with a low frequency of this genotype observed in patients with RA. Moreover, although no statistical significance was found between the groups, the presence of the G/A heterozygous genotype, which was more frequent in the control group, was associated with a protective factor for the disease.

In a study involving the SNP -308 G/A of the *TNFα* gene for chikungunya, Bucardo et al. [28], in a Nicaraguan population, revealed the presence of the G/A genotype as a protective factor against persistent joint pain by CHIKV, this genotype being less frequent in individuals with continuous arthralgia after infection compared to individuals who recovered more quickly. In turn, Fernandez-Mestre et al. [50], in an investigation of SNPs for dengue in a Venezuelan population, revealed the presence of the A allele of the SNP -308 G/A as a risk factor for dengue hemorrhagic fever, since this allele was significantly more frequent in patients with hemorrhagic manifestations than in individuals with classic symptoms of the disease. This finding supports studies that report a possible association between elevated levels of TNFα with vascular permeability and hemorrhage in patients with dengue hemorrhagic fever.

In summary, it is known that individual genetic variations have the capacity to influence the type of immune response triggered by the host upon an infection [30]. In this context, it is important to emphasize that the distinction in terms of ethnic and racial groups among world populations should be considered in studies involving the analysis of SNPs. The Brazilian population is representative of high genetic variability, resulting from intense miscegenation among peoples, characterized mainly by three parental populations: European, African, and Native American, noting that there are distinct estimates of ancestry even for populations within the same state [51].

Among the limitations of the study, it is noteworthy that clinical data were obtained based on the report provided by each patient, and that, being a retrospective study, the individuals might not reliably remember the symptomatologic aspects presented when the disease developed. Furthermore, due to the retrospective nature of the study, confirmation of CHIKF cases was specifically done through the detection of anti-CHIKV IgG antibodies, whereas anti-CHIKV IgM antibodies were not assessed. We emphasize the need for studies involving a larger sample size, especially for SNP analysis. In addition, the distinction between sample sizes in comparisons involving the ASY group may provide a low power of the association statistic.

## Figures and Tables

**Table 1:** Clinical manifestations in individuals who had chikungunya.

Clinical Manifestations	Number of Observations *n* = 102 (%)
Arthralgia	102 (100)
Fever	87 (85.3)
Myalgia	87 (85.3)
Headache	73 (71.6)
Edema	65 (63.7)
Prostration	53 (52)
Retro-orbital pain	42 (41.2)
Spots on the skin	39 (38.2)
Nausea	33 (32,3)
Fatigue	24 (23.5)
Abdominal pain	23 (22.5)
Dizziness	23 (22.5)
Back pain	22 (21.6)
Vomiting	22 (21.6)
Diarrhea	18 (17.6)
**Joints affected**	
Knees	77 (75.5)
Ankles	71 (69.6)
Elbow	67 (65.7)
Shoulders	54 (52.9)
Wrists	50 (49)
Hips	41 (40.2)

**Table 2:** Comparative analysis between sex and joint pain intensity in relation to CCA development.

Characteristics	Non-CCA	CCA	*p*	OR (95% CI)
	*n* = 29 (%)	*n* = 73 (%)		
**Sex (%)**				
Female	15 (51.7)	56 (76.7)	0.018	3.08 (1.29–7.41)
Male	14 (48.3)	17 (23.3)		
**Mild Pain**				
Yes	6 (20.7)	3 (4.1)	0.015	0.16 (0.04–0.63)
No	23 (79.3)	70 (95.9)		
**Moderate Pain**				
Yes	11 (37.9)	12 (16.4)	0.034	0.32 (0.12–0.89)
No	18 (62.1)	61 (83.6)		
**Severe Pain**				
Yes	7 (24.1)	15 (20.5)	0.791	0.81 (0.29–2.11)
No	22 (75.9)	58 (79.5)		
**Very Severe Pain**				
Yes	5 (17.2)	43 (58.9)	<0.001	6.88 (2.29–17.7)
No	24 (82.8)	30 (41.1)		

The *p* value was determined by Fisher’s exact test.

**Table 3:** Genotypic and allelic distribution in the case and control groups for the -174 G/C SNP in the *IL-6* gene.

*IL-6* -174 G/C	Case *n* = 102 (%)	Control *n* = 182 (%)	*p*	OR (95% CI)
**Genotypes**				
GG	56 (54.9)	87 (47.8)	-	(Reference)
GC	39 (38.2)	80 (43.9)	0.284	0.76 (0.45–1.25)
CC	7 (6.9)	15 (8.2)	0.509	0.72 (0.28–1.94)
GC + GG	95 (93.1)	167 (91.7)	0.563	0.88 (0.59–1.34)
GC + CC	46 (45.1)	95 (52.2)	0.250	0.75 (0.46–1.22)
**Alleles**				
G	151(74.0)	254 (69.8)	-	(Reference)
C	53 (26.0)	110 (30.2)	0.283	0.81 (0.55–1.18)

*p* value; OR, odds ration; CI, confidence interval. Statistical significance (*p* < 0.05).

**Table 4:** Genotypic and allelic distribution of case, control, and ASY groups for the -174 G/C SNP in the *IL-6* gene.

*IL-6* -174 G/C	ASY *n* = 29 (%)	Case *n* = 102 (%)	*p*	OR (95% CI)	ASY *n* = 29 (%)	Control *n* = 182 (%)	*p*	OR (95% CI)
**Genotypes**								
GG	17 (58.6)	56 (54.9)	-	(Reference)	17 (58.6)	87 (47.8)	-	(Reference)
GC	11 (37.9)	39 (38.2)	0.867	0.93 (0.40–2.16)	11 (37.9)	80 (43.9)	0.397	0.70 (0.33–1.64)
CC	1 (3.4)	7 (6.9)	0.676	0.47 (0.04–3.07)	1 (3.4)	15 (8.2)	0.461	0.34 (0.03–2.10)
GC + GG	28 (96.5)	95 (93.1)	0.932	0.97 (0.50–1.87)	28 (96.5)	167 (91.7)	0.647	0.86 (0.45–1.68)
GC + CC	12 (41.4)	46 (45.1)	0.722	0.86 (0.38–1.92)	12 (41.4)	95 (52.7)	0.279	0.65 (0.29–1.45)
**Alleles**								
G	45 (77.6)	151 (74.0)	-	(Reference)	45 (77.6)	254 (69.8)	-	(Reference)
C	13 (22.4)	53 (26.0)	0.580	0.82 (0.40–1.62)	13 (22.4)	110 (30.2)	0.224	0.67 (0.34–1.27)

*p* value; OR, odds ration; CI, confidence interval. Statistical significance (*p* < 0.05).

**Table 5:** Genotypic and allelic distribution in the case and control groups for the -308 G/A SNP in the *TNFα* gene.

*TNFα -308* G/A	Case *n* = 102 (%)	Control *n* = 182 (%)	*p*	OR (95% CI)
**Genotypes**				
GG	73 (71.6)	142 (78.0)	-	(Reference)
GA	29 (28.4)	39 (21.4)	0.193	1.45 (0.84–2.53)
AA	0 (0.0)	1 (0.5)	1.000	0.00
GA + GG	102 (100)	181 (99.4)	0.628	1.10 (0.75–1.58)
GA + AA	29 (28.4)	40 (22)	0.223	1.41 (0.82–2.46)
**Alleles**				
G	175 (85.8)	323 (88.7)	-	(Reference)
A	29 (14.2)	41 (11.3)	0.304	1.31 (0.79–2.15)

*p* value; OR, odds ration; CI, confidence interval. Statistical significance (*p* < 0.05).

**Table 6:** Genotypic and allelic distribution of case, control, and ASY groups for the -308 G/A SNP in the *TNFα* gene.

*TNFα* -308 G/A	ASY *n* = 29 (%)	Case *n* = 102 (%)	*p*	OR (95% CI)	ASY *n* = 29 (%)	Control *n* = 182 (%)	*p*	OR (95% CI)
**Genotypes**								
GG	23 (79.3)	73 (71.6)	-	(Reference)	23 (79.3)	142 (78.0)	-	(Reference)
GA	6 (20.7)	29 (28.4)	0.405	0.66 (0.24–1.78)	6 (20.7)	39 (21.4)	0.916	0.95 (0.37–2.39)
AA	0 (0.0)	0 (0.0)	1.000	0.00	0 (0.0)	1 (0.5)	1.000	0.00
GA + GG	29 (100)	102 (100)	0.747	0.90 (0.49–1.70)	29 (100)	181 (99.4)	0.971	0.99 (0.55–1.74)
GA + AA	6 (20.7)	29 (28.4)	0.405	0.66 (0.24–1.78)	6 (20.7)	40 (22.0)	0.876	0.93 (0.36–2.32)
**Alleles**								
G	52 (89.6)	175 (85.8)	-	(Reference)	52 (89.6)	323 (88.7)	-	(Reference)
A	6 (10.3)	29 (14.2)	0.444	0.70 (0.29–1.74)	6 (10.3)	41 (11.3)	0.836	0.91 (0.39–2.26)

*p* value; OR, odds ration; CI, confidence interval. Statistical significance (*p* < 0.05).

## Data Availability

The data presented in this study are available from the corresponding author upon reasonable request, as confidentiality agreements protect the privacy of research participants.

## Supplementary Materials

The following supporting information can be downloaded at: https://www.mdpi.com/article/10.3390/v17121543/s1, Table S1: Allelic association of SNP -308 G/A of the *TNFα* gene with joint pain in the cases group. Table S2: Allelic association of SNP -174 G/C of the *IL-6* gene with joint pain in the cases group. Table S3: Genotypic and allelic distribution of CCA, control and non-CCA groups for the -174 G/C SNP in the *IL-6* gene. Table S4: Genotypic and allelic distribution of CCA, control and non-CCA groups for the -308 G/A SNP in the *TNFα* gene.
