# Supplementary material for: Chronic Chikungunya Arthritis in Northeastern Brazil: An Association with Very Severe Joint Pain and Lack of Correlation with IL-6 and TNFα Gene Polymorphisms
Source: Viruses. 2025 Nov 26;17(12):1543. doi: 10.3390/v17121543 (PMC12737619; doi:10.3390/v17121543)
Supplement: Supplementary file 1 [file viruses-17-01543-s001.zip › viruses-3955520-supplementary.pdf]

Table S1 - Allelic association of SNP −308 G/A of the *TNFα* gene with joint pain in the cases group.

Joint Pain Intensity	Frequency N = 102 (%)	A carriers N = 29 (%)	Non-A-carriers N = 73 (%)	P-value
Very severe	48 (47.1)	16 (55.2)	32 (43.8)	0.308
Moderate	23 (22.5)	3 (10.3)	20 (27.4)	0.071
Severe	22 (21.6)	9 (31)	13 (17.8)	0.183
Mild	9 (8.8)	1 (3.4)	8 (11)	0.440

The P- value was determined by Fisher's Exact Test.

Table S2 - Allelic association of SNP −174 G/C of the *IL-6* gene with joint pain in the cases group.

Joint Pain Intensity	Frequency N = 102 (%)	C carriers N = 46 (%)	Non-C-carriers N = 56 (%)	P-value
Very severe	48 (47.1)	23 (50)	25 (44.6)	0.691
Moderate	23 (22.5)	12 (26.1)	11 (19.6)	0.482
Severe	22 (21.6)	9 (19.6)	13 (23.2)	0.810
Mild	9 (8.8)	2 (4.3)	7 (12.5)	0.180

The P- value was determined by Fisher's Exact Test.

Table S3 **-** Genotypic and allelic distribution of CCA, control and non-CCA groups for the -174 G/C SNP in the *IL-6* gene.

***IL-6* -174 G/C**	**CCA N= 73 (%)**	**Control N= 182 (%)**	***P***	**OR (95% CI)**	**CCA N = 73 (%)**	**Non-CCA N = 29 (%)**	***P***	**OR (95% CI)**
**Genotypes**								
GG	39 (53.4)	87 (47.8)	-	(Reference)	39 (53.4)	16 (55.2)	-	(Reference)
GC	30 (41.1)	80 (44)	0.535	0.84 (0.48 – 1.49)	30 (41.1)	10 (34.5)	0.658	1.23 (0.50 - 3.27)
CC	4 (5.5)	15 (8.2)	0.434	0.59 (0.20 – 1.87)	4 (5.5)	3 (10.3)	0.665	0.55 (0.14 - 2.39)
GC + GG	69 (94.5)	167 (91.7)	0.734	0.92 (0.57 – 1.49)	69 (94.5)	26 (89.7)	0.820	1.09 (0.51 - 2.32)
GC + CC	34 (46.6)	95 (52.2)	0.417	0.80 (0.46 – 1.37)	34 (46.6)	13 (44.8)	0.873	1.07 (0.45 - 2.45)
**Alleles**								
G	108 (74)	254 (69.8)	-	(Reference)	108 (74)	42 (72.4)	-	(Reference)
C	38 (26)	110 (30.2)	0.346	0.81 (0.52 – 1.26)	38 (26)	16 (27.6)	0.819	0.92 (0.46 - 1.79)

P value; OR, odds ration; CI, confidence interval. Statistical significance ($p < 0.05$).

Table S4 - Genotypic and allelic distribution of CCA, control and non-CCA groups for the -308 G/A SNP in the *TNFα* gene.

***TNFα* -308 G/A**	**CCA N= 73 (%)**	**Control N= 182 (%)**	***P***	**OR (95% CI)**	**CCA N = 73 (%)**	**Non-CCA N = 29 (%)**	***P***	**OR (95% CI)**
**Genotypes**								
GG	51 (69.9)	142 (78.0)	-	(Reference)	51 (69.9)	22 (75.9)	-	(Reference)
GA	22 (30.1)	39 (21.4)	0.147	1.57 (0.84 – 2.91)	22 (30.1)	7 (24.1)	0.544	1.36 (0.54 - 3.44)
AA	0 (0.0)	1 (0.5)	1.000	0.00	0 (0.0)	0 (0.0)	1.000	0.00
GA + GG	73 (100)	181 (99.4)	0.588	1.12 (0.74 – 1.71)	73 (100)	29 (100)	0.806	1.09 (0.57 - 2.13)
GA + AA	22 (30.1)	40 (22)	0.170	1.53 (0.83 – 2.83)	22 (30.1)	7 (24.1)	0.544	1.36 (0.54 - 3.44)
**Alleles**								
G	124 (84.9)	323 (88.7)	-	(Reference)	124 (84.9)	51 (87.9)	-	(Reference)
A	22 (15.1)	41 (11.3)	0.238	1.40 (0.79 – 2.40)	22 (15.1)	7 (12.1)	0.580	1.29 (0.55 – 3.34)

P value; OR, odds ration; CI, confidence interval. Statistical significance ($p < 0.05$).
